# Base excision repair imbalance in colorectal cancer has prognostic value and modulates response to chemotherapy

**DOI:** 10.18632/oncotarget.14909

**Published:** 2017-01-31

**Authors:** Natalia M. Leguisamo, Helena C. Gloria, Antonio N. Kalil, Talita V. Martins, Daniel B. Azambuja, Lisiane B. Meira, Jenifer Saffi

**Affiliations:** ^1^ Genetic Toxicology, Universidade Federal de Ciências da Saúde de Porto Alegre (UFCSPA), Porto Alegre, Rio Grande do Sul, Brazil; ^2^ Oncology and Colorectal Surgery, Santa Casa de Misericórdia de Porto Alegre (ISCMPA), Porto Alegre, Rio Grande do Sul, Brazil; ^3^ Department of Clinical and Experimental Medicine, Faculty of Health and Medical Sciences, University of Surrey, Guildford, United Kingdom

**Keywords:** colorectal cancer, base excision repair, prognosis, energy metabolism, temozolomide

## Abstract

Colorectal cancer (CRC) is prevalent worldwide, and treatment often involves surgery and genotoxic chemotherapy. DNA repair mechanisms, such as base excision repair (BER) and mismatch repair (MMR), may not only influence tumour characteristics and prognosis but also dictate chemotherapy response. Defective MMR contributes to chemoresistance in colorectal cancer. Moreover, BER affects cellular survival by repairing genotoxic base damage in a process that itself can disrupt metabolism. In this study, we characterized BER and MMR gene expression in colorectal tumours and the association between this repair profile with patients’ clinical and pathological features. In addition, we exploited the possible mechanisms underlying the association between altered DNA repair, metabolism and response to chemotherapy. Seventy pairs of sporadic colorectal tumour samples and adjacent non-tumour mucosal specimens were assessed for BER and MMR gene and protein expression and their association with pathological and clinical features. MMR-deficient colon cancer cells (HCT116) transiently overexpressing *MPG* or *XRCC1* were treated with 5-FU or TMZ and evaluated for viability and metabolic intermediate levels. Increase in BER gene and protein expression is associated with more aggressive tumour features and poor pathological outcomes in CRC. However, tumours with reduced MMR gene expression also displayed low *MPG*, *OGG1* and *PARP1* expression. Imbalancing BER by overexpression of *MPG*, but not *XRCC1*, sensitises MMR-deficient colon cancer cells to 5-FU and TMZ and leads to ATP depletion and lactate accumulation. *MPG* overexpression alters DNA repair and metabolism and is a potential strategy to overcome 5-FU chemotherapeutic resistance in MMR-deficient CRC.

## INTRODUCTION

Colorectal carcinoma (CRC) is the third most common malignancy worldwide, and over 1 million new cases of colorectal carcinoma are diagnosed each year [1]. Disease prognosis and therapy selection for CRC largely depends on pathology-related staging following the tumour-node-metastasis (TNM) classification and staging system. Despite widely used, TNM staging seldom predicts the clinical outcome of CRC patients accurately. Highlighting the limitations of the TNM classification system is the observation that 20-40% of patients with relatively low-grade, stage II colorectal cancer rapidly worsen and die [2, 3]. Moreover, despite significant advances in diagnosis and treatment, CRC-related mortality remains unchanged for the past 20 years and patients with advanced disease derive limited if any benefit from the recent advances in adjuvant therapy [4].

Therapy for CRC includes surgery, radiation and/or chemotherapeutic schemes, largely based on the use of 5-fluorouracil (5-FU) and its prodrug capecitabine [5]. Treatment with 5-FU results in thymidylate synthase (TS) inhibition, depletion of thymidine triphosphates (TTPs) available for DNA synthesis, and misincorporation of FdUTP (5-FdUrd triphosphate) into DNA, and/or of FUTP (5-fluorouridine triphosphate) into RNA [5]. Despite therapy, cancer recurrence is common within the first few years following treatment completion, indicating stem cells repopulation [6] and drug resistance [7].

Base excision repair (BER) and mismatch repair (MMR) both play a key role in dictating cellular responses to 5-FU treatment, since BER recognizes and removes uracil and 5-FU from DNA [8] and MMR drives 5-FU-induced cytotoxicity [9]. Greater survival benefit after 5-FU treatment is observed in patients whose tumours are MMR-proficient while MMR-deficiency, characterising 15-20% of sporadic CRC cases, is not associated with survival benefit after 5-FU treatment [10]. In the case of BER, published evidence suggests an association between inappropriate BER and increased tumour aggressiveness in CRC [for review see 11]. However, how exactly BER affects tumour malignancy remains to be determined since conflicting results are often reported on the expression of a limited number of BER genes [12, 13, 14]. Despite these limitations, BER modulation is under active investigation as a potential therapeutic approach for chemotherapy sensitization [for review see 15].

BER is a multi-step repair pathway acting on damaged bases generated by alkylation, oxidation or deamination and proceeds through a sequence of reactions depending on the initial base lesion [11]. BER is initiated upon base removal by one of many substrate-specific DNA glycosylases, such as N-methylpurine-DNA glycosylase (MPG) and 8-oxoguanine-DNA glycosylase (OGG1) [11]. The enzymatic steps taking place once BER is initiated lead to the generation of toxic intermediates such as apurinic/apyrimidinic (AP) sites and single strand breaks (SSB). Efficient BER completion is coordinated by an important BER accessory factor, the chromatin associated enzyme poly(ADP-ribose) polymerase or PARP. PARP is activated upon binding to sites of SSB and recruits downstream BER enzymes for completion of the repair process [16].

BER modulation may affect cellular outcomes to chemotherapy depending on the step in the pathway that is modulated. For example, preventing BER initiation by loss of DNA glycosylase activity may, in some instances, protect against the detrimental consequences of cytotoxic BER intermediate accumulation [for review see 17]. Conversely, BER imbalance by overexpression of an initiating glycosylase such as MPG sensitizes cancer cells to chemotherapeutic alkylating drugs [18]. In addition, once BER is initiated, PARP status can also affect cellular responses to chemotherapy [17]. Indeed, PARP inhibitors have emerged as a potential therapeutic strategy to increase sensitivity to chemotherapy by alkylating agents [19]. However, because PARP activation at SSB can also lead to cytotoxicity via glycolysis inhibition and energy depletion [20, 21, 22], disrupting energy metabolism through chemotherapy and BER modulation coupled to the remarkable dependence of cancer cells on aerobic glycolysis for energy production [23, 24] can represent a powerful approach in cancer treatment [25].

In this study, we profiled BER and MMR gene expression in sporadic colorectal tumours and matched non-tumour tissues, and related the expression of these DNA repair genes with clinical tumour features and pathological staging. We find that high BER gene expression is associated with poor clinical outcomes. In addition, we overexpressed the BER proteins MPG and XRCC1 in MMR-defective HCT116 cells and assessed how increased levels of these proteins affected cell survival and metabolic intermediates after 5-FU and TMZ treatments. We find that MPG overexpression, but not XRCC1 overexpression, results in increased 5-FU and TMZ sensitivity, ATP depletion and lactate accumulation. Our results suggest that BER modulation can alter both tumour metabolism and response to DNA damage, making it an attractive therapeutic target for colorectal cancer.

## RESULTS

### BER imbalance and reduction in MGMT expression characterize colorectal cancer tissues

**Figure 1:**
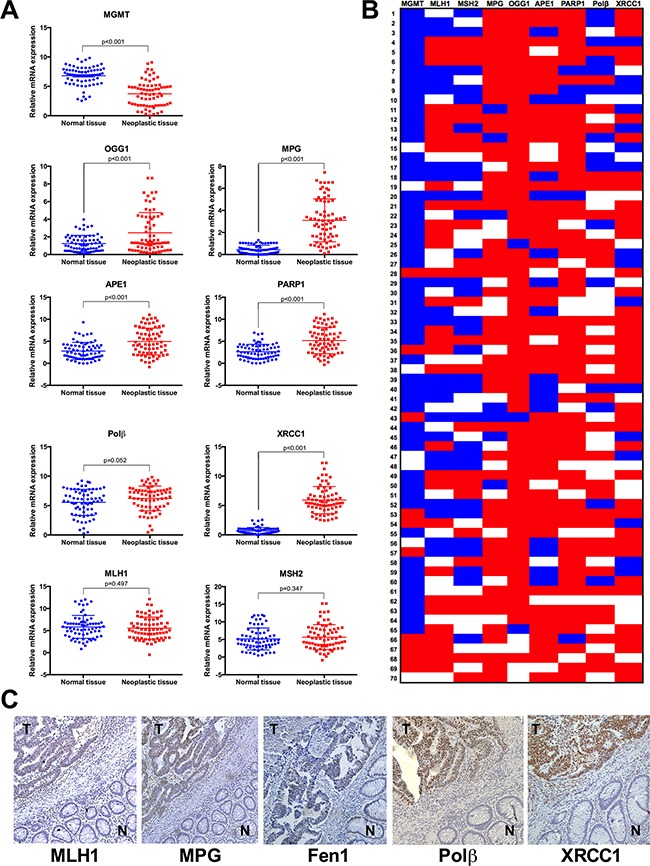
Sporadic colorectal tumours present imbalance of BER genes in comparison to healthy adjacent mucosa **A**. Gene expression was quantified for a panel of DNA repair genes by real-time qPCR analysis in neoplastic and normal mucosal tissues from 70 patients with sporadic colorectal cancer. The following genes were examined: MGMT, MLH1, MSH2, MPG, OGG1, APE1, PARP1, Polβ and XRCC1. Gene expression data are shown as scatter diagrams. **B**. Heat map of individual gene expression changes in sporadic colorectal cancer. Fold changes were calculated for neoplastic tissue *vs*. adjacent normal tissue. Blue indicates decreased relative gene expression, red indicates increased relative gene expression and white indicates no change in gene expression. **C**. Representative photomicrographs showing immunohistochemical staining in colorectal tumour specimens for MLH1, MPG, Fen1, Polβ and XRCC1; N: normal tissue; T: tumour tissue. Images were taken at x200 magnification.

The clinicopathological characteristics of the 70 patients are shown in Table 1. The mRNA levels for MGMT (direct repair); MLH1 and MSH2 (MMR); and OGG1, MPG, APE1, PARP1, Polβ and XRCC1 (BER) were first quantified using qPCR in 70 pairs of primary sporadic colorectal tumours and adjacent healthy mucosal tissues. Gene expression data for the whole patient cohort is shown in Figure 1A. In addition, a diagram showing the relative expression of four BER genes in paired samples of colorectal tumours and adjacent non-tumour samples from 25 patients with high expression for both *MPG* and *XRCC1* genes is shown in Supplementary Figure 1.

**Table 1:** Clinicopathological features of CRC patients included in this study (n=70)

Variable	n (%)
**Total cases**	70
**Age** (mean± SD)	67.7± 11
**Age, y**	
≤65	30 (43)
>65	40 (57)
**Gender**	
Male	38 (54)
Female	32 (26)
**Tumour location**	
Colon	42 (60)
Rectum	28 (40)
**Histology**	
Adenocarcinoma	60 (85)
Mucinous	10 (15)
**Cellular differentiation**	
Well or moderately differentiated	30 (43)
Poorly differentiated	40 (57)
**Tumour invasive depth**	
T1-T2	20 (29)
T3-T4	50 (71)
**Lymph node status**	
N0 (n=0)	19 (27)
N1 (n≤3)	26 (37)
N2 (n>3)	15 (21)
**Lymphatic invasion**	
No	38 (54)
Yes	32 (46)
**Perineural invasion**	
No	44 (63)
Yes	26 (37)
**Preoperative CEA, ng/mL**	
≤5	42 (60)
>5	28 (40)
**AJCC/TNM stage**	
I-II	41 (59)
III	29 (41)

The numbers in parentheses indicate the percentage of cases with a specific clinical or pathological characteristic.

MGMT mRNA levels were reduced in tumour samples in comparison to healthy mucosal tissues (Figure 1A), and this reduction was seen in 73% of patients (Figure 1B). MGMT promoter methylation and consequently decreased expression is observed in 27-40% of chemo resistant metastatic colorectal tumours and is a frequent event in colorectal carcinogenesis [26]. However, since MGMT loss has been also identified in healthy colorectal tissue, this event has been referred as a “field defect”, which means it is neither necessary nor sufficient to cancer progression [27].

Differently from direct repair, BER is multi-step repair process that requires coordinated expression of several proteins to prevent accumulation of cytotoxic repair intermediates. With the exception of Polβ, where we observed no significant differences in gene expression levels between tumour and healthy tissues, all other BER genes examined were found to be overexpressed in tumour colorectal tissues if compared to healthy tissue (Figure 1A). However, it is important to note that a consistent upregulation for the entire group of BER genes was only observed in approximately 7% of our patient cohort (5 out of 70 patients). This means that a majority of sporadic CRC cases present an imbalance in this pathway, with some BER genes overexpressed without compensatory changes in downstream BER steps (Figure 1B).

**Figure 2:**
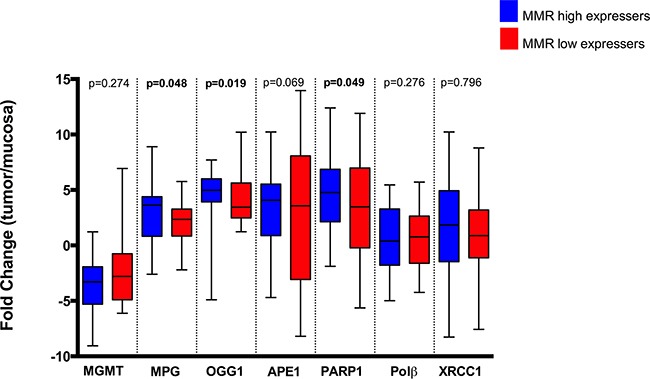
MMR status (low or high gene expression of MLH1 and MSH2 simultaneously) correlation with MLH1, MGMT, Fen1, Polβ and XRCC1 fold change (tumour/normal tissue) Statistically significant differences (p<0.05) are highlighted in bold font.

Regarding MMR, we found no significant difference in expression levels for MLH1 and MSH2 between tumour and control tissues (Figure 1A). This was intriguing since MLH1 inactivation was reported to occur in 15-20% of colorectal tumours [28]. With this in mind, we then analysed expression data for individual patients, and observed that 31% of all patients showed a reduction in MLH1 expression in tumour tissue in comparison to healthy tissue (log_2_fold change < -1) and 24% of patients displayed reduced expression of both MMR genes in neoplastic tissue (Figure 1B). It is well established that a functioning MMR pathway is necessary for the triggering of apoptosis in response to mispairing 5-FU induced base damage [29], and in the absence of MMR, the repair of such damage will require the BER pathway. Additionally, MMR deficiency is associated with better prognosis for colorectal cancer patients [30] but if this is associated to a particular BER status is currently not known. For this reason, we assessed a potential association between BER gene expression profiles and MMR status (Figure 2). Sample classification was based on expression levels for both MLH1 and MSH2: samples with lower expression in tumour vs normal tissue for both genes were considered as MMR deficient, all other samples were designated MMR proficient (including patients with higher or similar expression in tumour vs. normal tissue). This analysis showed that deficiency in MMR (characterized by low *MLH1* and *MSH2* gene expression simultaneously) correlates with lower expression of MPG, OGG1 and PARP1, but not other BER proteins. While the biological relevance of this correlation is unclear, it raises the interesting possibility that this particular profile would help explain the better prognosis seen in MMR deficient colorectal cancer patients [30].

Next, we assessed protein expression levels by immunohistochemistry for MLH1, MPG, FEN1, Polβ and XRCC1 in tumour vs. healthy tissue of this patient cohort (Figure 1C and Supplementary Figure 2). Higher protein expression levels in tumour tissue were identified for MLH1, MPG, Polβ and XRCC1, respectively, in 79%, 59%, 56% and 57% of all cases (Supplementary Table 1). Of note, gene expression data positively correlates with protein expression for MLH1: r=0.699, p<0.001; MPG: r=0.7126, p<0.0001; Polb: r=0.3838, p=0.016 and XRCC1: r= 0.6641, p<0.0001.

In addition, because BER imbalance may modulate cell survival in a replication-dependent manner [31], we assessed whether overexpression of BER proteins, notably MPG, was associated with higher tumour proliferation by examining proliferating cell nuclear antigen (PCNA) expression in our samples. PCNA was overexpressed in 51 patients (69%) and it was positively correlated with MPG (r=0.2640, p=0.027) and Polβ (r=0.2833, p=0.017), but no correlation was found between PCNA and XRCC1 (r=-0.064, p=0.8388) (Supplementary Figure 3).

### Changes in BER gene and protein expression have prognostic value in patients with colorectal cancer

**Figure 3:**
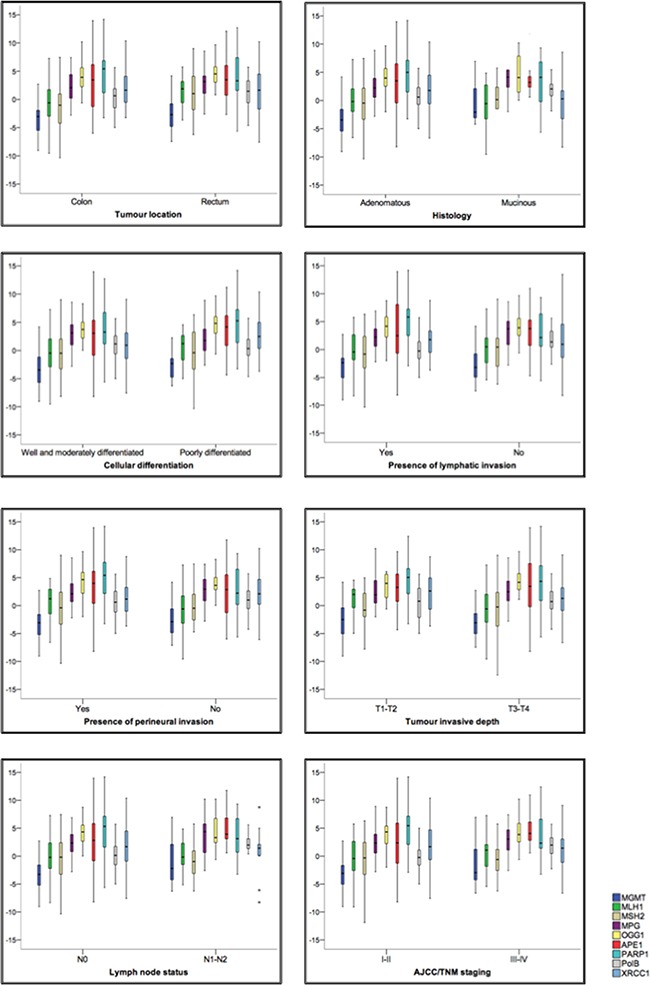
Expression changes in direct repair, MMR and BER genes are associated with specific clinical and pathological outcomes in CRC patients **A**. Correlations between gene expression and known clinical and pathological parameters were determined; statistical significance was assessed by correlating MMR status and BER and direct repair gene expression. Statistically significant gene expression correlates of clinical and pathological features are highlighted. **B**. Box plots showing fold change in gene expression between tumour tissue and normal adjacent mucosa (y axis). Horizontal lines represent the median; the bottom and top of the boxes represent the 25th and 75th percentiles, respectively; and the vertical bars represent the range of data.

Using regression-correlation analysis (Table 2), we next assessed whether the gene expression levels for the above proteins acting in the three distinct DNA repair pathways (namely, direct repair, MMR and BER) were associated with clinical and pathological features currently used for staging and prognosis determination in colorectal cancer patients (Figure 3A and 3B). We find that, primary tumours located in rectum are associated with higher expression for both *MLH1* (p=0.014) and *MSH2* (p=0.015). Our analysis also showed that more aggressive mucinous histological subtypes are associated with higher levels of *MGMT* (p=0.027) and *MPG* (p=0.031) expression. Poorly differentiated tumours were associated with increased *MPG* (p=0.033) and *XRCC1* (p=0.042) expression. Presence of lymphatic and/or perineural invasion is associated with overexpression of *MPG* (p=0.044 for lymphatic invasion), *PARP1* (p=0.016 and 0.043, for lymphatic and perineural invasion, respectively), but also with reduction of *Polβ* gene expression (p=0.024 and 0.003, for lymphatic and perineural invasion, respectively). Association between TNM stage III-IV and high *APE1* expression (p=0.018) and low *Polβ* expression (p=0.001) was also found. Finally, we found correlation between perioperative CEA (carcinoembryonic antigen) <5ng/ml and reduction of *MSH2* gene expression (p=0.033).

**Table 2:** Correlations between gene expression and known clinical and pathological parameters were determined

	MGMT	MLH1	MSH2	MPG	OGG1	APE1	PARP1	Polβ	XRCC1
**Age**	0.518	0.147	0.585	0.441	0.226	0.888	0.377	0.522	0.819
**Tumour location**	0.369	**0.014**	**0.015**	0.276	0.601	0.502	0.915	0.298	0.745
**Histology**	**0.027**	0.666	0.678	**0.031**	0.816	0.862	0.495	0.201	**0.042**
**Cellular differentiation**	0.176	0.233	0.949	**0.033**	**0.042**	0.302	0.444	0.321	**0.031**
**Tumour invasive depth**	0.812	**0.020**	0.743	0.832	0.302	0.704	0.709	0.733	0.317
**Lymph node status**	0.125	0.848	0.537	0.164	0.983	0.188	0.439	**0.003**	0.345
**Lymphatic invasion**	0.884	0.766	0.687	**0.044**	0.889	0.847	**0.016**	**0.024**	0.451
**Perineural invasion**	0.555	0.062	0.852	0.459	0.395	0.406	**0.043**	**0.003**	0.389
**Preoperative CEA**	0.472	0.103	**0.033**	0.253	0.704	0.392	0.652	0.247	0.892
**TNM stage**	0.198	0.732	0.953	0.131	0.920	**0.018**	0.329	**0.001**	0.576

Associations of DNA repair gene expression with clinical parameters were evaluated using multiple linear regression analysis. Statistically significant gene expression correlates of clinical and pathological features are highlighted (p<0.05).

We also verified the associations between clinical and pathological characteristics and protein expression (Supplementary Table 1). Overexpression of MLH1 is associated with tumour location (p=0.002) and tumour invasive depth (p=0.045); high expression of MPG is associated with tumour location (p=0.014), lymphatic invasion (p=0.006) and TNM stage (p=0.008); overexpression of Polβ is associated with perineural invasion (p=0.043) and advanced TNM staging (p=0.008); XRCC1 overexpression is associated with histology (p=0.044) and presence of lymphatic invasion (p=0.005); while FEN1 protein expression did not present any significant association with clinical data.

### Overexpression of MPG, but not XRCC1, sensitises colon cancer cells to 5-FU and temozolomide

Since we found that colorectal tumours that present increased MPG and XRCC1 mRNA and protein levels are more prone to unfavourable pathological outcomes, we decided to investigate *in vitro* the potential impact of modulating the levels of these two key BER components on cellular viability after exposure to 5-FU and TMZ. Human cells overexpressing MPG are more sensitive to alkylation-induced damage in a Polβ-dependent manner [19], but no evidence was yet reported for MPG and Polβ levels affecting the cellular response to 5-FU. While 5-FU is a classic first line chemotherapeutic drug in CRC treatment not known to induce MPG substrates, TMZ is an alkylating agent inducing MPG substrates (reviewed in 17]., presently under investigation for metastatic CRC patients heavily pre-treated but non-responders (Clinical Trials identifiers: NCT01051596, NCT02414009).

**Figure 4:**
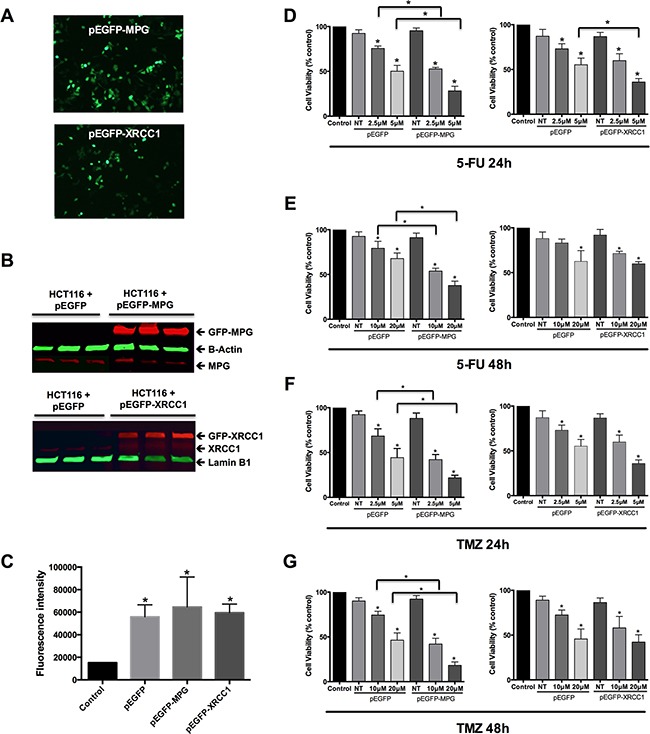
Overexpression of MPG, but not of XRCC1, sensitizes colon cancer cells to 5-FU and TMZ **A**. Representative photomicrographs of viable HCT116 cells displaying normal cellular morphology and exhibiting green GFP fluorescence 48h after transfection. **B**. Western blot analysis of MPG and XRCC1 protein levels in HCT116 cells 48 hours after transfection. GFP-MPG protein: 64kD; β-Actin: 42kD; MPG: 37kD; GFP-XRCC1: 112kD; XRCC1: 85kD; Lamin-B1: 66kD. **C**. GFP fluorescence intensity 48h after transfection with the different GFP expressing vector constructs. **D**. and **E**. Viability assessment of cells overexpressing GFP alone, GFP-MPG fusion protein and GFP-XRCC1 fusion protein 24h (D) and 48h (E) after treatment with 5-FU. **F**. and **G**. Viability assessment of cells overexpressing GFP alone, GFP-MPG fusion protein and GFP-XRCC1 fusion protein 24h (**F**.) and 48h (**G**.) after treatment with TMZ. Symbols above the error bars represent statistically significant difference (p<0.05) between cells overexpressing GFP alone and cells overexpressing the GFP-fusion proteins.

We treated colorectal tumour HCT116 cells transiently overexpressing MPG or XRCC1 (Figure 4A, 4B and 4C) with 5-FU (2.5μM and 5μM) (Figure 4D and 4E) and with TMZ (10μM and 20μM) for 24h and 48h (Figure 4F and 4G). Expression levels of these exogenous proteins were monitored by western blotting and shown to be similar in the different replicate experiments (Figure 4B). The above-mentioned doses correspond to the IC_20_ and IC_50_, respectively, for each drug (Supplementary Figure 4). The MPG overexpressing colon cancer cells were more sensitive to both 5-FU doses after 24h and 48h in comparison to XRCC1-overexpressed cells (p<0.001). XRCC1 overexpression did not significantly affect cell viability to 5-FU, while MPG overexpression led to a significant reduction in cellular viability after 5-FU treatment (p<0.01), with viability reduced to 31% or 21% 24h or 48h after treatment, respectively. The same scenario was observed after the TMZ IC_50_ treatment: the overexpression of MPG, but not of XRCC1, promoted an increase in cellular sensitivity to TMZ in both time points, and viability was reduced to 28% or 15% 24h or 48h after treatment, respectively (p<0.01 for both time points).

### MPG overexpression increases cytosolic ATP levels and TMZ treatment leads to ATP depletion in colon cancer cells

In order to understand the mechanisms underlying the increased sensitivity to cytotoxic drugs that MPG - but not XRCC1 - overexpression promotes in colon cancer cells, we considered the possibility that imbalancing BER by MPG overexpression could induce metabolic dysfunction, such as ATP loss and lactate accumulation. Surprisingly, MPG-overexpressing cells presented a 2-fold increase in cytosolic ATP basal levels (48h after the transfection) in comparison to control (p<0.001) and to the XRCC1-overexpressed cells (p<0.01), but no basal changes in lactate secretion (Supplementary Figure 5).

**Figure 5:**
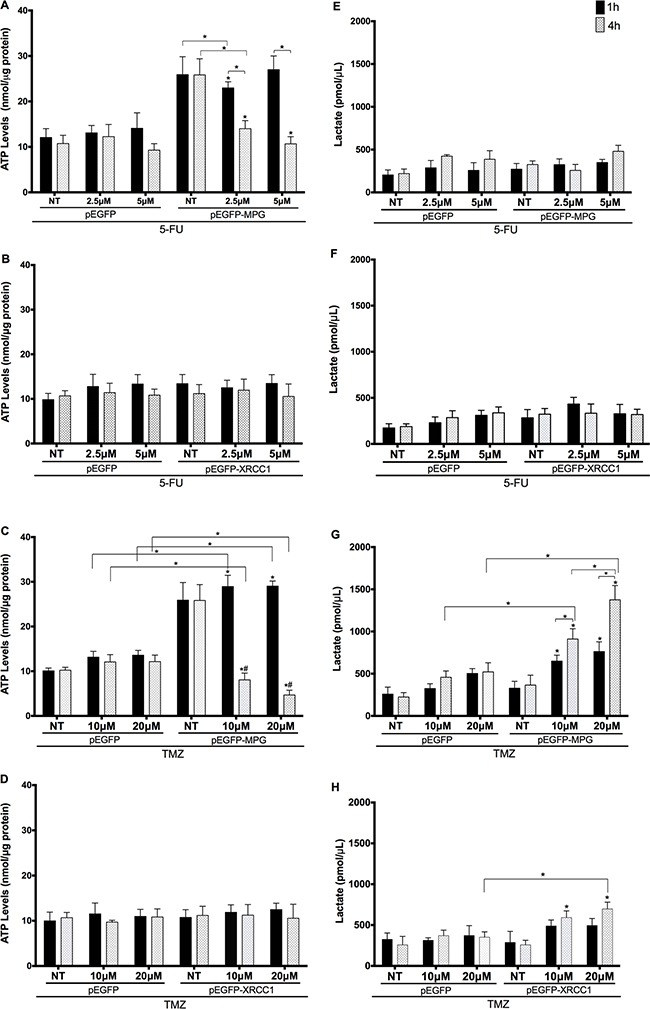
ATP levels are modulated by MPG overexpression and 5-FU and TMZ treatments **A**. and **B**. Cytosolic ATP levels in MPG and XRCC1-overexpressing cells treated with 5-FU; **C** and **D**. Cytosolic ATP levels in MPG and XRCC1-overexpressed cells treated with TMZ; **E**. and **F**. lactate secretion levels in MPG and XRCC1- overexpressed cells treated with 5-FU; **G**. and **H**. lactate secretion levels in MPG and XRCC1- overexpressed cells treated with 5-TMZ. Symbols above the error bars represent statistically significant differences (p<0.01) in comparison to the cells transfected with the same plasmid that did not receive treatment. (#) Represents statistically significant differences (p<0.01) comparing two time points after treatment. NT: non-treated cells. Comparisons between two data sets (empty plasmid *vs*. plasmid for overexpression) are indicated individually (*p<0.01).

We then treated cells with 5-FU and TMZ and assessed these parameters one and four hours post-treatment (Figure 5). ATP levels were significantly reduced 1 h after 5-FU treatment (2.5μM) in MPG-overexpressing colon cancer cells, but the basal increase resulting from MPG overexpression meant 5-FU-treated cells still maintained higher ATP levels than control. However, after 4h, ATP levels were further reduced, and reached control levels (Figure 5A). Similarly, TMZ treatment led to even greater changes in ATP levels in MPG-overexpressing cells (Figure 5C). While ATP levels increased 1h after treatment, ATP levels dramatically dropped 4h after treatment, becoming even lower than controls. In counterpart, we did not observe any changes in ATP levels following 5-FU and TMZ treatments in cells overexpressing XRCC1 (Figure 5B and 5D).

While 5-FU treatment did not affect lactate secretion levels in either genotype (Figure 5F and 5G), lactate levels were altered by TMZ treatment. We observed a substantial time and dose-dependent increase in lactate accumulation in cells overexpressing MPG (Figure 5G) and a modest one in the XRCC1-overexpressed cells after 4h (Figure 5H).

## DISCUSSION

We investigated if alterations in BER, MMR and direct repair pathways are associated with a particular clinical outcome in CRC and therefore have prognostic value. Here, we report that BER genes are upregulated in a majority of sporadic CRC, represented by increases in MPG, OGG1, APE1, PARP1 and XRCC1 expression. We also report the association between these individual repair gene expression levels and poor outcome features in colorectal tumours. However, when we analysed the entire BER pathway as a whole for each individual CRC case, we noticed that there was no consistent levels of co-expression for the BER proteins, indicating an imbalance or deregulation in this repair mechanism.

The classical relevance of DNA repair in sporadic CRC is attributed to MMR inactivation, by *MLH1* and *MSH2* loss or silencing [30], which occurs in 15-20% of the patients. While this characteristic is considered to promote better overall survival rate and modulate the response to 5-FU [32], individual patients with advanced tumour staging still present poor overall response rates (20–30%) [2]. Presently, however, DNA repair in CRC emerges as a promising strategy for personalized treatment. 5-FU-induced DNA damage is repaired by both MMR and BER, and it has been suggested that a crosstalk between these DNA repair pathways modulates cytotoxicity and resistance [32]. Thus, a potential approach to enhance the DNA-based mechanisms of 5-FU-mediated cell death is to identify how BER alterations can contribute to cytotoxicity.

While coordinated BER pathway avoids excessive toxic repair intermediate formation, such as AP sites and SSBs, and contributes to tumour resistance and lethal outcomes for the patient [11], imbalances in different steps of this pathway (*e.g*., caused by overexpression, loss or inhibition) are responsible for a wide range of cellular fates. We found that overexpression of BER genes was associated with clinical features that indicate tumour aggressiveness, such as poor cellular differentiation, presence of lymphatic and perineural invasion and advanced tumour stage. However, our analysis revealed that the BER pathway is likely to be imbalanced in our patient cohort (we define BER imbalance as tumour where at least three of six BER genes are altered) and we propose BER imbalance contributes to increased tumour aggressiveness in these patients. In support of this idea, modulation of individual BER enzymes levels was shown to influence several phenotypes both *in vivo* and *in vitro*. For example, DNA glycosylase inhibition jeopardizes BER initiation and results in accumulation of cytotoxic and mutagenic base lesions [33]; on the other hand, while overexpression of Polβ increases spontaneous mutagenesis in mammalian cells [34], Polβ downregulation leads to hypersensitivity to alkylation-induced DNA damage [35]. Yet, XRCC1-deficient mammalian cells do not properly repair SSBs due to inefficient DNA termini clean up and nick ligation [36].

The expression status of a full panel of BER genes had not been previously evaluated in CRC patients, and it is presently unclear to what magnitude MMR and BER collectively contribute to 5-FU and TMZ cellular sensitivity. Using colon cancer HCT116 cells, which are MMR-deficient (h*MLH1*^-/-^ and h*MSH3*^-/-^), wild type for *TP53* and MGMT-proficient [37]. we were able to show that MPG overexpression results in increased sensitization to 5-FU in colon cancer cells, despite 5-FU not being a known MPG substrate. MPG overexpression was previously reported in some types of neoplasias, including CRC [38, 39], however, to our knowledge, MPG-dependent sensitization to 5-FU in colon cancer cells has not been previously reported. On the other hand, previous reports showed that disrupting BER by XRCC1 depletion did not sensitize cells to 5-FU [40]. That XRCC1 modulation does not affect response to 5-FU is striking given its prominent role in later steps of BER. One possible explanation might be altered sub-pathway choice; long-patch BER may complete repair downstream of APE1-mediated incision in an XRCC1-independent fashion. Further investigation is warranted to assess whether 5-FU-induced damage is repaired by long–patch BER, bypassing the requirement for short-patch BER.

Interestingly, we find that MPG, but not XRCC1, overexpression resulted in greater sensitivity to TMZ in colon cancer cells. MPG overexpression was previously associated with cellular sensitisation to alkylation: studies involving breast, glioma, and ovarian cancer cell lines reported that MPG overexpression conferred sensitivity to alkylating agents, such as TMZ [19, 38]. MPG expression was also previously shown to predict temozolomide sensitivity in glioblastoma and ovarian cancer cell lines [16], but TMZ sensitivity in CRC was not previously addressed. Clinical trials for TMZ in CRC were designed to exploit the association between MGMT deficiency and increased sensitivity to temozolomide [41, 42]. Indeed, MGMT promoter hypermethylation and low MGMT expression appear to be early events in CRC patients [43], consistently with our clinical results. In addition, concomitant downregulation of both MGMT and MPG correlate with an improved response to TMZ [44]. Thus, if higher MPG levels lead to BER imbalance and a better response to TMZ, we suggest TMZ treatment would potentially benefit CRC patients and BER imbalance could be used as a new potential biomarker for this emerging CRC therapy.

MPG overexpression sensitized cells to 5-FU and TMZ, leading to increased ATP depletion and lactate accumulation upon treatment with these agents. However, while 5-FU treatment reduced basal ATP levels to control levels’ but had no effect in lactate accumulation, TMZ treatment led to a dramatic reduction in ATP levels associated with significant increase in lactate secretion, a scenario compatible to lactic acidosis. A similar metabolic dysfunction was already reported in several pathophysiological conditions, such as ischemia and diabetes [45, 46]. Indeed, in a streptozotocin-induced diabetes model, pancreatic beta-cell death was mediated by Parp-1 activation [47] and by Mpg-initiated BER [48], molecular events known to contribute to tissue damage by depleting cells of free energy [20]. In addition, lactate accumulation within tumour tissue is chiefly due to the increased glycolytic rate of cancer cells, and it also correlates with metastasis and poor disease-free and overall survival [49]. Moreover, cell death can also be mediated by repression of glycolytic genes as a cellular response to lactate accumulation, which is associated with significantly increased patient survival rates [50]. Collectively, these results suggest that TMZ-induced ATP depletion and high lactate may indeed represent a viable option to modulate patient outcomes to therapy.

Our data suggest that BER imbalance characterises sporadic CRC and influences tumour aggressiveness and potentially patient outcome. We propose that it would be clinically important to assess MPG expression levels in sporadic colorectal cancer cases. MPG overexpression could increase 5-FU therapeutic efficacy so the identification of MPG overexpressing tumours could indicate a better response to therapy in these cancer patients.

**Figure 6:**
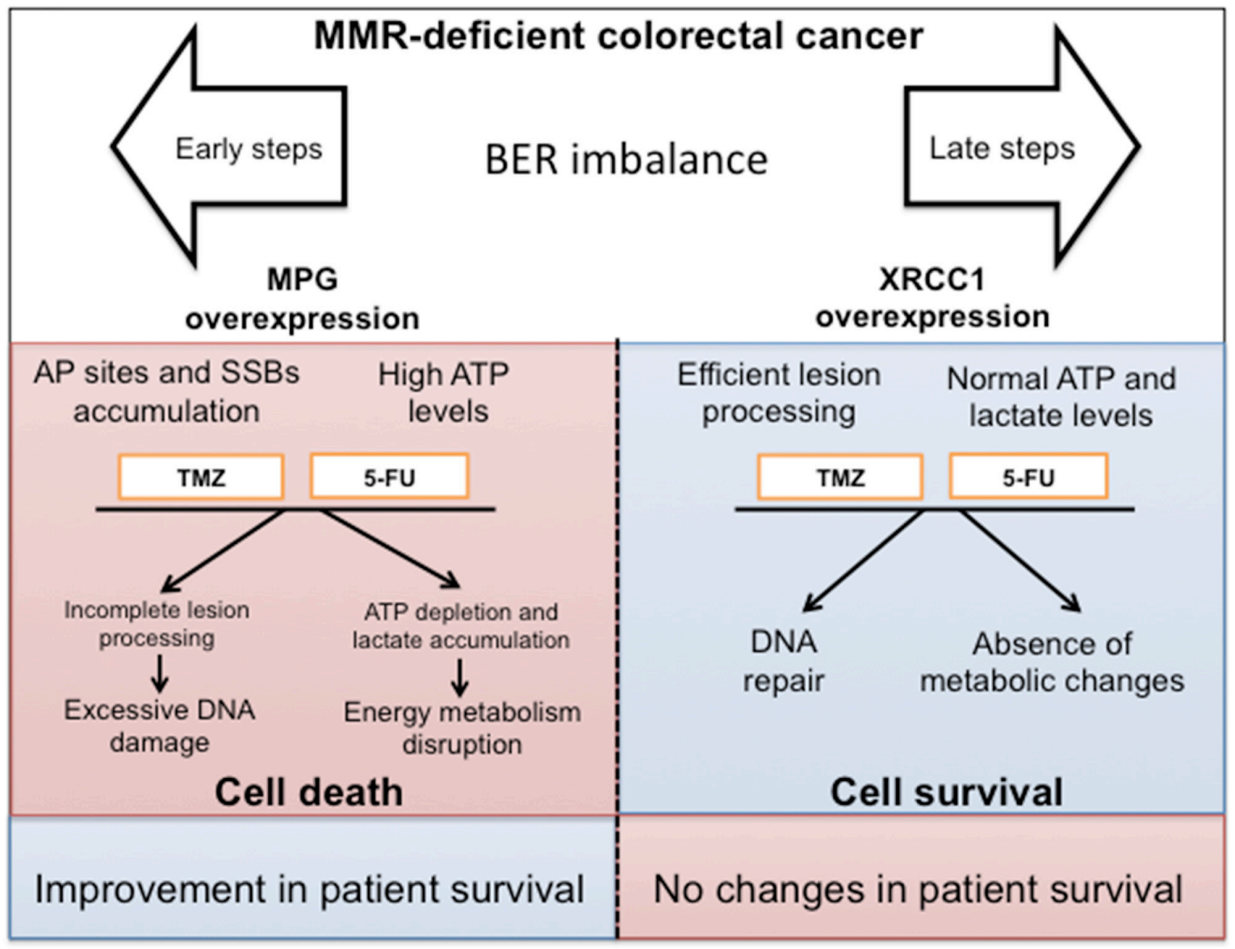
Model depicting the potential therapeutic value of BER status assessment in colorectal cancer BER pathway imbalance can be therapeutically exploited to benefit MMR-deficient colorectal cancer patients. MMR-deficient sporadic CRC patients derive little benefit from chemotherapy, Modulation of early BER steps (i.e. MPG overexpression) results in enhanced sensitivity to DNA damage and energy metabolism disruption, representing therefore an attractive therapeutic target for this subset of CRC patients.

We propose a model to depict how imbalances in early (such as MPG) or late (such as XRCC1) BER steps can alter therapeutic outcomes in CRC (Figure 6). Our model proposes that the metabolic characteristics of tumour cells contribute to tumour aggressiveness but can also be exploited to improve chemotherapeutic response. BER imbalance in initial steps (such as through MPG overexpression) leads to increased sensitivity to 5-FU and TMZ treatments and energy metabolism disruption. On the other hand, imbalancing BER at later steps (e.g. XRCC1 overexpression) would not significantly alter tumour response to 5-FU or TMZ, since no energy metabolism disruption was observed in XRCC1 overexpressing cells upon 5-FU or TMZ treatment.

In light of our results, we propose TMZ is an attractive candidate for a new chemotherapy approach in CRC. Our findings in HCT116 cells, which are MGMT proficient and MMR deficient, support the idea that TMZ-induced N-methylpurines rather than *O*^6^-methylguanine adducts significantly contribute to cell death in MMR-deficient cells. TMZ may be especially effective in MMR-deficient MPG-overexpressing colon cancer cells, concomitantly inducing DNA damage and disrupting metabolism, potentially leading to higher therapeutic efficiency for this subset of CRC patients.

## MATERIALS AND METHODS

### Patients

A total of 70 surgically resected fresh tissue specimens comprising of tumour tissues (percentage of tumour cells >70%) and adjacent normal tumour-free regions (non tumour healthy mucosa at >10 cm distance from the tumour) of primary sporadic CRC were collected between 2013 and 2015. Only cases with a primary diagnosis of colorectal cancer undergoing upfront surgery were selected for the purpose of this study. We excluded patients who had received pre-operative treatment in the form of radiation or chemotherapy. We also excluded patients with family history of Familiar Adenomatous Polyposis (FAP) and Hereditary Non-polyposis Colorectal Cancer (HNPCC). Histopathological grades and clinical staging were evaluated according to the standard criteria described in the sixth edition of AJCC/UICC Classification by pathologists blinded for the study purposes. Ethical committees of participating institutions approved the study, and written informed consent was obtained from patients before study enrolment. The study was carried out in accordance with the principles of the Helsinki Declaration.

### Quantitative reverse transcription PCR

We evaluated the differential gene expression in colorectal tumours and healthy paired tissues in duplicate by RT2 Profiler™ PCR Array (SABiosciences/Qiagen). The selected genes composing the arrays were: *MPG, OGG1, APEX1, PARP1, Polβ* and *XRCC1* from the BER pathway; *MLH1* and *MSH2* from the MMR pathway; and the direct repair gene *MGMT*.

Regions of high tumour cellularity (minimum of 80%) were selected for RNA extraction. RNA was extracted and purified from 30 mg of both fresh tumours and adjacent mucosa samples using an RNeasy mini kit (SABiosciences/Qiagen) according to the manufacturer's instructions. cDNA was synthesized from 1 μg of total RNA using the RT^2^ PCR Array First Strand Kit (SABiosciences/Qiagen) according to supplier's recommendations. Reaction was prepared using RT2 SYBR-Green/Rox PCR Master Mix (SABiosciences/Qiagen). Data analysis was based on the ΔΔCT method with normalization of the raw data to two housekeeping genes (*EIF2B* and *PPIA*).

### Immunohistochemistry

MLH1, MPG, FEN1, POLβ and XRCC1 expression in primary tumours was examined from formalin-fixed paraffin-embedded samples. Tissues were sectioned at 4-μm and mounted on silanized slides, which were stored at 4°C. After deparaffinization and rehydration, the sections were quenched with 3% H_2_O_2_ in methanol to block endogenous peroxidase. Bovine serum albumin at 5% (BSA) was then applied to prevent non-specific binding. The sections were incubated with anti-MPG (dilution 1:100, Abcam, mouse, EPR10959(B)), anti-Polβ (dilution 1:500, Abcam, rabbit, ab26343), anti-FEN1 (dilution 1:800, Abcam, rabbit, ab17993), anti-XRCC1 (dilution 1:50, Abcam, mouse, ab1838), anti-MLH1 (dilution 1:100, Abcam, ab92312) and PCNA (dilution: 1:400, mouse, #2586) antibodies, and then incubated with appropriate secondary antibodies (DAKO). Diaminobenzidine (DAB) was used as chromogen and the sections were counterstained with haematoxylin. Omission of the primary antibody was used as a negative control. Protein expression was evaluated using Quick Score (QS) (by assessing both staining intensity and the percentage of the stained area with a given intensity) and dichotomized in low (score between 0 and 4) and high expression (score between 5 and 12) (Supplementary Material and Methods). Only stained nuclei of malignant cells were assessed.

### Cell culture and transfection

The human colorectal cancer cell line HCT116 was obtained from the American Type Culture Collection and cultured in McCoy's 5A Medium (Gibco) containing 10% FBS (Sigma), penicillin (10^7^ U/L) and streptomycin (10 mg/L), maintained at 37°C in a humidified 5% CO_2_ incubator. The HCT116 cell line was authenticated at the DNA Diagnostics Center (DDC) from Public Health England (PHE) using short tandem repeat (STR) methodology and used within 5 months of thawing a frozen vial. The plasmid allowing for the overexpression of a GFP-MPG fusion protein was a kind gift from Clara F. Charlier in the Meira lab, while the GFP-XRCC1 fusion construct was a kind gift from Dr Akira Yasui. For transient transfections, cells were seeded into 96-well plates at 1×10^4^ cells per well. After 24 hours, cells were transfected with Lipofectamine 2000 following the manufacturer's protocol (Invitrogen). After 5 hours of incubation at 37°C, the transfection medium was replaced with fresh complete medium. All experiments were performed in triplicate and 48h after transfection. Basal values correspond to the 48h post-transfection time point but without intervention.

### Cytotoxic drugs treatment

5-FU and TMZ were purchased from Sigma-Aldrich and reconstituted in DMSO, aliquoted and maintained at -20°C. 48h after transfection, chemotherapeutic agents at the desired concentrations were added into each well and plates were incubated for 24h and 48h (for viability assays) or for 1h and 4h (for metabolic measurements) in an incubator with 5% CO_2_ at 37°C.

### Cell viability assay

Viability was assessed with the CellTiter-Aqueous MTS reagent (Promega), 24 and 48 h post-treatment. Briefly, MTS reagent was added into each well and incubated for 3h with 5% CO_2_ at 37°C. MTS absorbance was then measured at 490nm. Results are reported as the percentage of treated cells relative to the cells without treatment (% Control).

### Western blot

Cell lysates were obtained with mammalian cell lysis buffer M-PER (PIERCE) containing protease inhibitors (Sigma). Protein content was determined with the BCA Protein Assay according to manufacturer's instructions (Pierce, Thermo Fisher Scientific). Twenty micrograms of protein lysate was loaded onto a SDS-PAGE gel (Bio-Rad) and transferred eletrophoretically onto a polyvinylidene fluoride membrane. The membranes were blocked overnight with PBS containing 0.1% Tween 20 in 5% skim milk at 4°C and subsequently probed using the following primary antibodies: anti-MPG (1:250, Sigma, HPA006531), anti-XRCC1 (1:500, Abcam, ab1838), anti-β-Actin (1:2000, Abcam, mouse ab6276 and rabbit ab52614) and anti-Lamin-B1 (1:2000, mouse ab8983 and rabbit ab133741). Detection followed by incubation with IRDye® 800CW Goat anti-Rabbit IgG or IRDye 680RD Goat anti-Mouse IgG (both from LI-COR). Membranes were scanned using an Odyssey CLx infrared imaging system (LI-COR Biosciences).

### ATP and lactate measurements

For lactate measurements, medium was sampled from cells 1h and 4h after treatment and deproteinized with Amicon centrifugal filters (Merck Millipore) for lactate dehydrogenase removal. Colorimetric method was performed with lactate assay kit (Sigma-Aldrich) following the manufacturer's instructions. Absorbance was read at 570 nm.

For ATP measurements, cells were lysed with digitonin-based buffer (Cayman). Intracellular ATP was determined by a luciferin/luciferase method using an ATP bioluminescent assay kit (Sigma-Aldrich). Luminescence was measured using a 96-well plate luminometer. Cytosolic ATP content was calculated by an ATP standard curve and normalized to cellular protein content/well.

### Statistical analysis

Clinical data was analysed using SPSS version 22.0 (SPSS Inc.). Qualitative variables were compared using the χ2 Test and Fisher's exact test; and quantitative variables were analysed by the *t* test. Univariate analysis of clinical factors including age, sex, TNM stage, tumour invasion, lymph node metastases, cellular differentiation, location and tumour size was assessed by the log-rank test. Experimental data was analysed with GraphPad Prism 6 software (GraphPad) and statistical analysis carried out by two-way ANOVA and Tukey's post-hoc. The results were considered statistically significant when p-value was less than 0.05.

## SUPPLEMENTARY MATERIALS FIGURES AND TABLES




