# Supplementary material for: Base excision repair imbalance in colorectal cancer has prognostic value and modulates response to chemotherapy
Source: Oncotarget. 2017 Jan 31;8(33):54199–214. doi: 10.18632/oncotarget.14909 (PMC5589573; doi:10.18632/oncotarget.14909)
Supplement: Supplementary file 1 [file oncotarget-08-54199-s001.pdf]

Base excision repair imbalance in colorectal cancer has prognostic value and modulates response to chemotherapy

SUPPLEMENTARY MATERIALS

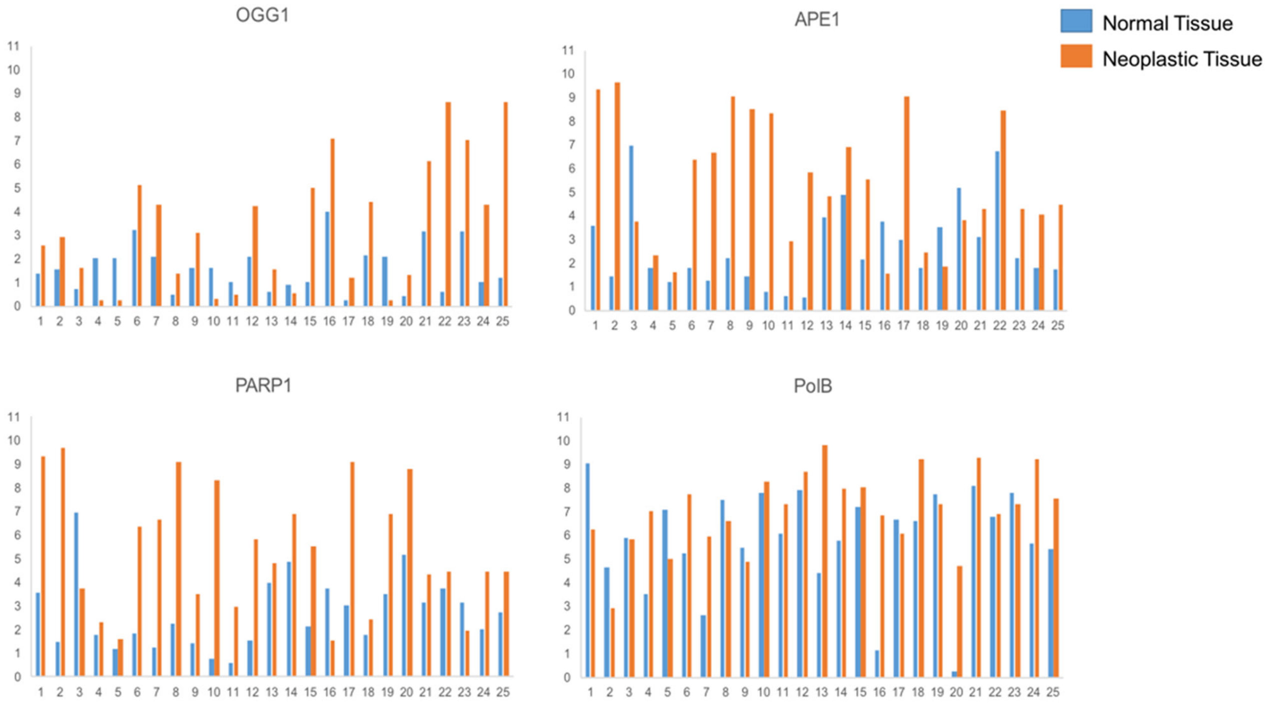

Supplementary Figure 1: Diagram showing the gene expression levels of OGG1, APE1, PARP1 and Polβ of CRC from patients that presented both MPG and XRCC1 overexpressed in tumour tissues (n=25).

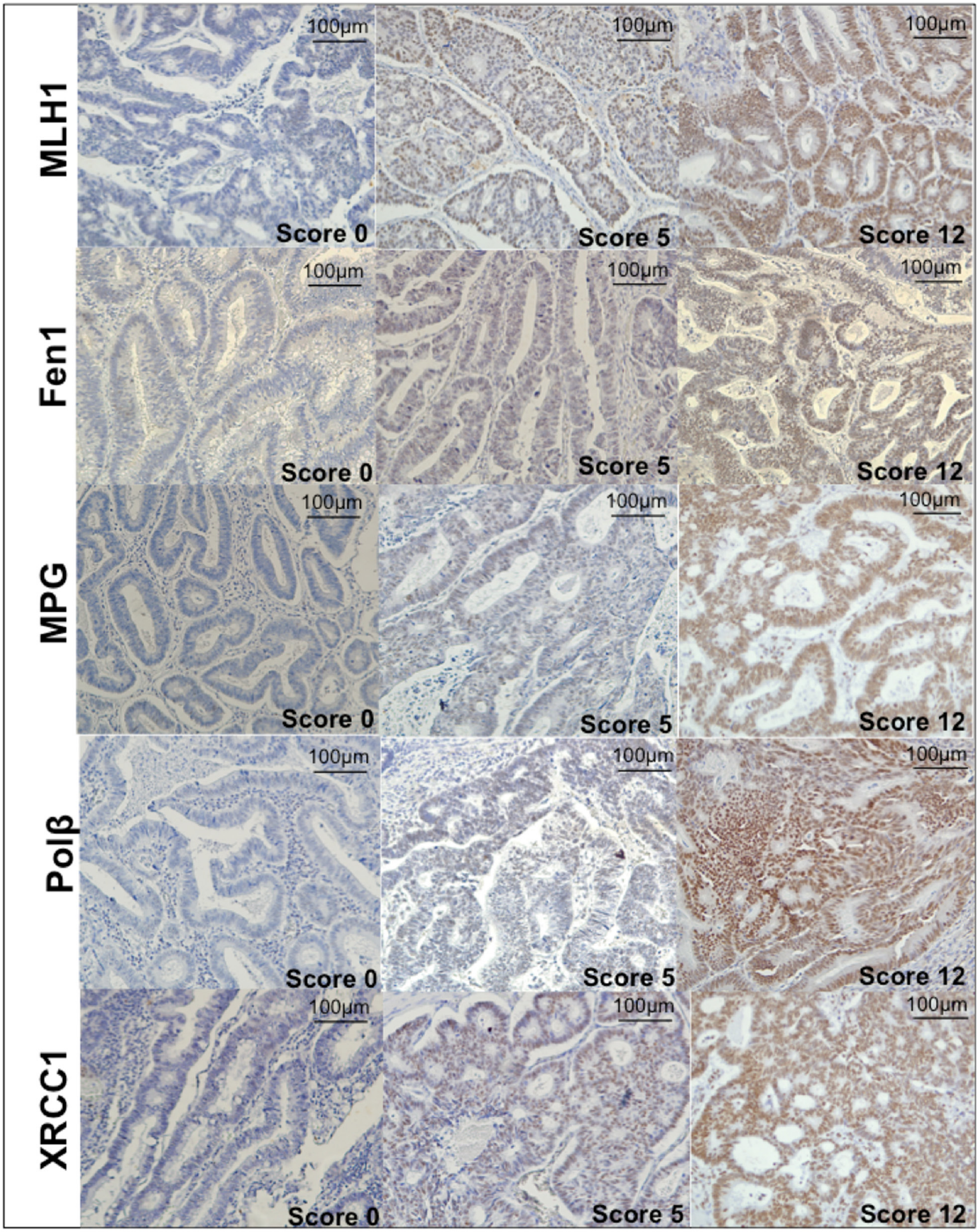

Supplementary Figure 2: Immunohistochemistry images representative of the scores calculated for MLH1, MPG, Fen1, Polβ and XRCC1 protein expression in colorectal tumour tissue specimens. Magnification is x100.

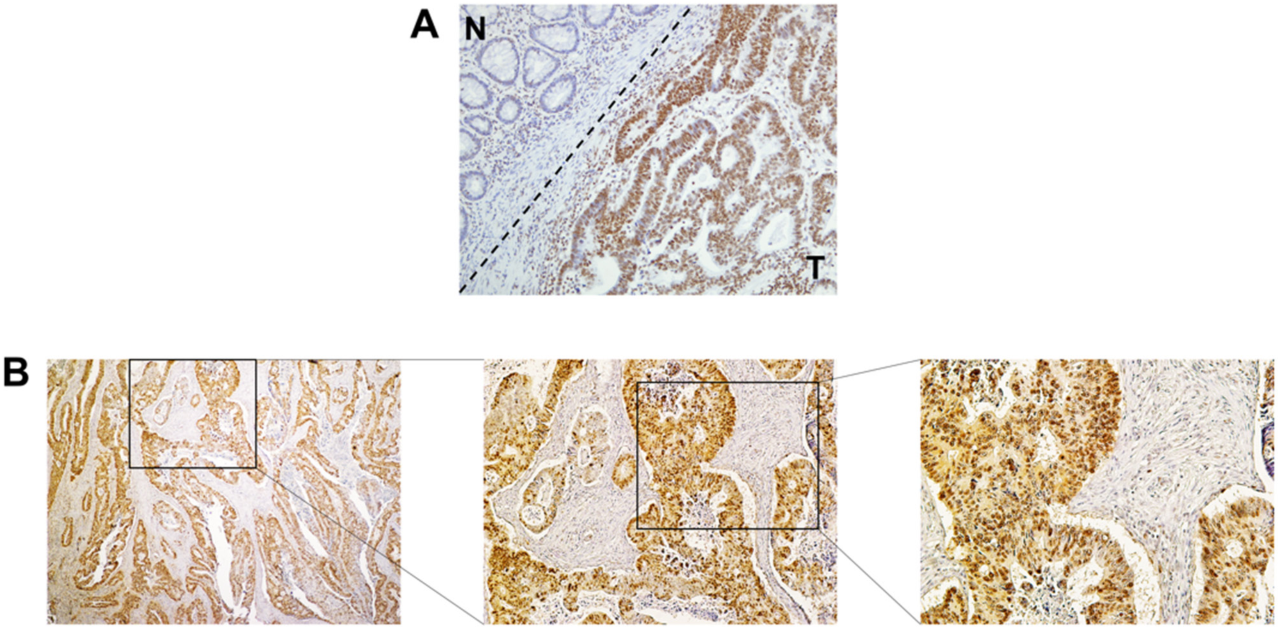

Supplementary Figure 3: A. Representative photomicrographs showing immunohistochemical staining in colorectal tumour specimens and healthy tissues. N: normal tissue; T: tumour tissue. Images were taken at x200 magnification. **B.** Representative images of PCNA positive protein expression in colorectal tumours. Magnification is x100, x200 and x400 respectively.

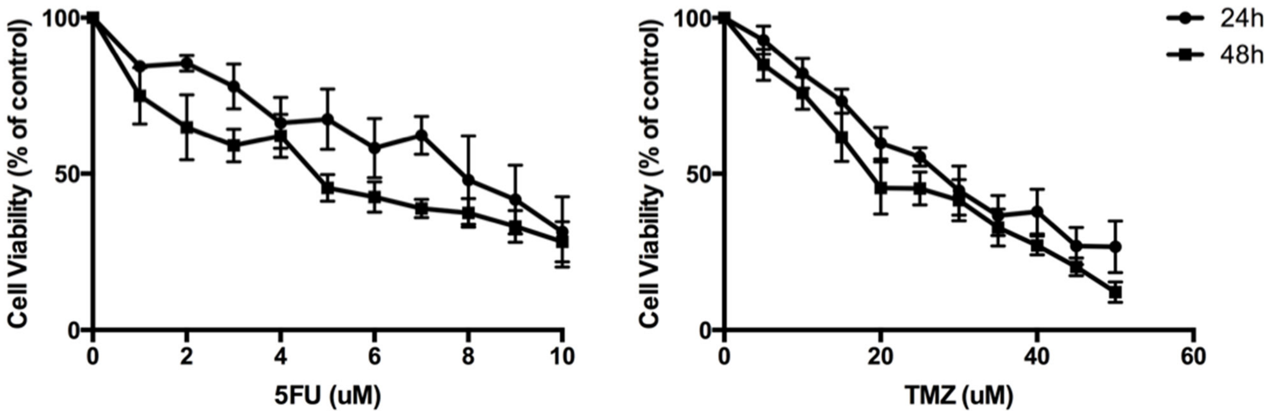

Supplementary Figure 4: Dose-response curves for IC_{20} and IC_{50} calculation of 5-FU and TMZ treatments used in viability and metabolic assays.

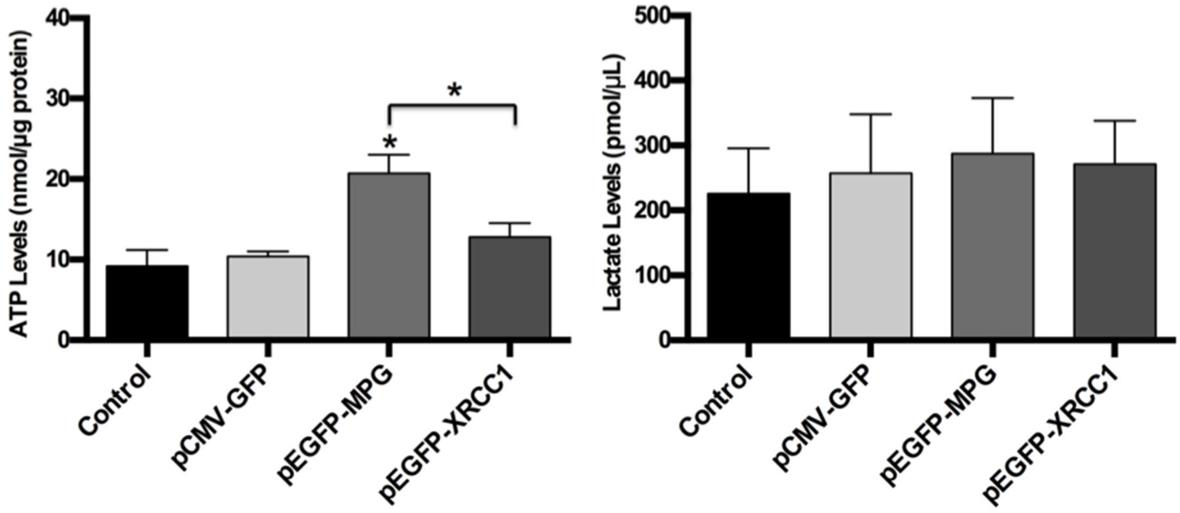

Supplementary Figure 5: Basal cytosolic ATP and extracellular lactate levels 48h after transfection of HCT116 cells with empty, MPG- or XRCC1-expression vectors. *$p<0.01$.

Supplementary Table 1: Clinicopathological findings and correlation with MLH1, MPG, Fen1, Pol and XRCC1 expression in colorectal tumour tissues. The "*" symbol denotes statistical significance. The numbers in parentheses indicate the percentage of tumours with a specific clinical or pathological feature for a protein expression subtype.

See Suplementary File 1
