# Supplementary material for: Base excision repair imbalance in colorectal cancer has prognostic value and modulates response to chemotherapy
Source: Oncotarget. 2017 Jan 31;8(33):54199–214. doi: 10.18632/oncotarget.14909 (PMC5589573; doi:10.18632/oncotarget.14909)
Supplement: Supplementary file 2 [file oncotarget-08-54199-s002.doc]

| **Supplementary Table 1: Clinicopathological findings and correlation with MLH1, MPG, Fen1, Pol and XRCC1 expression in colorectal tumour tissues.** The “*” symbol denotes statistical significance. The numbers in parentheses indicate the percentage of tumours with a specific clinical or pathological feature for a protein expression subtype. | | | | | | | | | | | | | | | | |
| --- | --- | --- | --- | --- | --- | --- | --- | --- | --- | --- | --- | --- | --- | --- | --- | --- |
| **Variable** |  | **MLH1** | | | **MPG** | | | **Fen1** | | | **Polβ** | | | **XRCC1** | | |
|  |  | **High** | **Low** | ***p Value*** | **High** | **Low** | ***p Value*** | **High** | **Low** | ***p Value*** | **High** | **Low** | ***p Value*** | **High** | **Low** | ***p Value*** |
| **Total cases** | 70 | 55 (79) | 15 (21) |  | 41 (59) | 29 (41) |  | 31 (44) | 39 (56) |  | 39 (56) | 31 (44) |  | 40 (57) | 30 (43) |  |
| **Age, years** |  |  |  |  |  |  |  |  |  |  |  |  |  |  |  |  |
| ≤65 | 30 (43) | 23 (77) | 7 (23) | *0.737* | 23 (77) | 7 (23) | *0.834* | 15 (50) | 15 (50) | *0.405* | 17 (47) | 13 (43) | *0.891* | 22 (73) | 8 (27) | *0.676* |
| >65 | 40 (57) | 32 (80) | 8 (20) |  | 18 (45) | 22 (55) |  | 16 (40) | 24 (60) |  | 22 (55) | 18 (45) |  | 18 (45) | 22 (55) |  |
| **Tumour location** |  |  |  |  |  |  |  |  |  |  |  |  |  |  |  |  |
| Colon | 42 (60) | **32 (76)** | **10 (34)** | ***0.002**** | 23 | 19 | *0.014* | 18 (43) | 24 (57) | *0.768* | 23 (55) | 19 (45) | *0.844* | 23 (55) | 19 (45) | *0.622* |
| Rectum | 28 (40) | **23 (82)** | **5 (18)** |  | 7 | 21 |  | 13 (46) | 15 (54) |  | 16 (57) | 12 (43) |  | 17 (61) | 11 (39) |  |
| **Histology** |  |  |  |  |  |  |  |  |  |  |  |  |  |  |  |  |
| Adenocarcinoma | 60 (85) | 47 (78) | 13 (22) | *0.905* | 34 (57) | 26 (43) | *0.428* | 27 (45) | 33 (55) | *0.768* | 35 (59) | 25 (41) | *0.281* | 34 (57) | 26 (43) | *0.844* |
| Mucinous | 10 (15) | 8 (80) | 2 (2) |  | 7 (70) | 3 (3) |  | 4 (40) | 6 (60) |  | 4 (40) | 6 (60) |  | 6 (60) | 4 (40) |  |
| **Cellular differentiation** |  |  |  |  |  |  |  |  |  |  |  |  |  |  |  |  |
| Well or moderately differentiated | 30 (43) | 23 (78) | 7 (22) | *0.355* | 16 (53) | 14 (47) | *0.441* | 7 (23) | 23 (77) | *0.728* | 22 (73) | 8 (27) | *0.891* | **14 (46)** | **16 (55)** | ***0.05**** |
| Poorly differentiated | 40 (57) | 22 (55) | 8 (45) |  | 25 (62) | 15 (38) |  | 24 (60) | 16 (40) |  | 17 (42) | 23 (58) |  | **26 (65)** | **14 (35)** |  |
| **Tumour invasive depth** |  |  |  |  |  |  |  |  |  |  |  |  |  |  |  |  |
| T1-T2 | 20 (29) | **19 (95)** | **1 (5)** | ***0.045**** | **16 (90)** | **4 (20)** | ***0.021**** | 10 (50) | 10 (50) | *0.543* | 10 (50) | 10 (50) | *0.543* | 12 (60) | 8 (40) | *0.760* |
| T3-T4 | 50 (71) | **36 (72)** | **14 (28)** |  | **25 (50)** | **25 (50)** |  | 21 (42) | 29 (58) |  | 29 (58) | 21 (42) |  | 28 (56) | 22 (44) |  |
| **Lymph node status** |  |  |  |  |  |  |  |  |  |  |  |  |  |  |  |  |
| N0-N1 | 55 (79) | 44 (80) | 11 (20) | *0.577* | 35 (67) | 20 (33) | *0.100* | 26 (47) | 29 (53) | *0.335* | 31 (56) | 24 (44) | *0.834* | 30 (54) | 25 (46) | *0.400* |
| N2 | 15 (21) | 11 (73) | 4 (27) |  | 6 (40) | 9 (60) |  | 5 (33) | 10 (67) |  | 8 (53) | 7 (47) |  | 10 (67) | 5 (33) |  |
| **Lymphatic invasion** |  |  |  |  |  |  |  |  |  |  |  |  |  |  |  |  |
| No | 38 (54) | 30 (79) | 8 (21) | *0.933* | **27 (71)** | **11 (29)** | ***0.021**** | 17 (45) | 21 (55) | *0.934* | 20 (53) | 18 (47) | *0.572* | **18 (47)** | **20 (53)** | ***0.042**** |
| Yes | 32 (46) | 25 (78) | 7 (22) |  | **14 (44)** | **18 (56)** |  | 14 (44) | 18 (56) |  | 19 (59) | 13 (41) |  | **22 (68)** | **10 (32)** |  |
| **Perineural invasion** |  |  |  |  |  |  |  |  |  |  |  |  |  |  |  |  |
| No | 44 (63) | 34 (77) | 10 (23) | *0.731* | 28 (64) | 16 (36) | *0.263* | 18 (41) | 26 (59) | *0.459* | **19 (43)** | **25 (57)** | ***0.006**** | 25 (57) | 19 (43) | *0.943* |
| Yes | 26 (37) | 21 (80) | 5 (20) |  | 13 (50) | 13 (50) |  | 13 (50) | 13 (50) |  | **20 (77)** | **6 (23)** |  | 15 (57) | 11 (43) |  |
| **Preoperative CEA, ng/mL** |  |  |  |  |  |  |  |  |  |  |  |  |  |  |  |  |
| ≤5 | 42 (60) | 33 (78) | 9 (22) | *1.000* | 26 (62) | 16 (38) | *0.488* | 19 (45) | 23 (55) | *0.844* | 22 (52) | 20 (48) | *0.492* | 25 (60) | 17 (40) | *0.622* |
| >5 | 28 (40) | 22 (78) | 6 (22) |  | 15 (53) | 13 (47) |  | 12 (43) | 16 (57) |  | 17 (61) | 11 (39) |  | 15 (53) | 13 (47) |  |
| **AJCC/TNM stage** |  |  |  |  |  |  |  |  |  |  |  |  |  |  |  |  |
| I-II | 41 (59) | 33 (80) | 8 (20) | *0.642* | **30 (73)** | **11 (27)** | ***0.003**** | 19 (47) | 22 (53) | *0.681* | **11 (27)** | **30 (63)** | ***0.008**** | 23 (56) | 18 (44) | *0.843* |
| III | 29 (41) | 22 (76) | 7 (24) |  | **11 (38)** | **18 (62)** |  | 12 (41) | 17 (59) |  | **28 (97)** | **1 (3)** |  | 17 (59) | 12 (41) |  |
